# Concurrent VVOR and VORS profiling reveals a threshold-dependent VOR suppression deficit in vestibular migraine

**DOI:** 10.3389/fneur.2026.1872711

**Published:** 2026-06-17

**Authors:** Huimin Fan, Jing Feng, Lipeng Cai, Qi Kong, Zhaohui Song, Pan Gu, Yuchuan Ding, Xiaokun Geng

**Affiliations:** 1 Department of Neurology and the Stroke Intervention and Translational Center (SITC), Beijing Luhe Hospital, Capital Medical University, Beijing, China; 2 Department of Neurosurgery, Wayne State University School of Medicine, Detroit, MI, United States; 3 China-America Institute of Neuroscience, Beijing Luhe Hospital, Capital Medical University, Beijing, China

**Keywords:** cerebellum, vestibular migraine, vestibulo-ocular reflex suppression (VORS), visually enhanced vestibulo-ocular reflex (VVOR), visual-vestibular integration

## Abstract

**Objectives:**

To define the dual profile of visual-vestibular integration in vestibular migraine (VM) by concurrently evaluating visually enhanced vestibulo-ocular reflex (VVOR) and vestibulo-ocular reflex suppression (VORS), and to determine which metric more closely reflects disease-related central vestibular dysfunction.

**Methods:**

In this retrospective case–control study, 57 patients with VM diagnosed according to the 2022 Bárány Society/ICHD-3 criteria and 30 healthy controls underwent VVOR and VORS testing using video-oculography during passive horizontal head rotation (0.5 Hz, ±15°). Clinical data were collected from medical records. Group differences in VVOR and VORS gains were analyzed, restricted cubic spline (RCS) regression combined with binary logistic regression was used to analyze the dose–response relationship between these indices and VM risk after adjusting for potential confounders.

**Results:**

Compared with controls, patients with VM showed significantly higher VVOR gains (mean: 1.4 ± 0.2 vs. 1.2 ± 0.2, *p* = 0.003) and markedly elevated VORS gains (0.5 ± 0.2 vs. 0.2 ± 0.1, *p* < 0.001). VVOR demonstrated a linear relationship with VM risk (nonlinearity *p* = 0.5629), with risk increasing gradually above 1.32 but high VVOR (≥1.32) was not independently associated with VM (OR = 0.44, 95% CI: 0.13–1.45, *p* = 0.2). In contrast, VORS showed a significant nonlinear S-shaped association with VM risk (nonlinearity *p* = 0.0001), showing the steepest rise in risk between 0.39 to 0.60 and a plateau thereafter. A high VORS gain (≥0.39) was strongly associated with VM (OR = 0.01, 95% CI: 0.001–0.08, *p* < 0.001), identifying VORS impairment as the more clinically relevant abnormality.

**Conclusion:**

This study provides the first simultaneous characterization of the enhancement and suppression arms of visual-vestibular integration in VM. The key contribution is the identification of a threshold-dependent VORS suppression deficit as a more specific indicator of VM-related central dysfunction, consistent with impaired cerebellar inhibitory control, whereas elevated VVOR appears to reflect a less specific adaptive response. These findings refine the physiological understanding of VM and support VORS-based stratification as a potential framework for mechanistic subtyping and targeted vestibular rehabilitation.

## Introduction

Vestibular migraine (VM) is the second most common cause of recurrent vertigo (1). Frequent attacks substantially impair daily functioning and are associated with elevated risks of anxiety, depression, and insomnia (2). However, the pathophysiology of VM remains poorly elucidated, and current management remains largely symptomatic, with limited efficacy (3).

Accumulating evidence points to central visual-vestibular integration dysfunction involving the cerebellum and brainstem, rather than isolated peripheral impairment (4). However, Conventional vestibular function tests have limited capacity to assess central integration. For instance, the VOR, an automatic, vision-independent reflex, primarily reflects brainstem and vestibular nerve function, not central integration (5). Conversely, tests that do engage central pathways-like smooth pursuit and optokinetic responses-often yield inconsistent results (6–8), limiting their clinical utility.

Visual-vestibular integration comprises two complementary components: the visually enhanced vestibulo-ocular reflex (VVOR) and VOR suppression (VORS) (9). VVOR refers to the enhancement of the VOR response during head rotation using external visual cues to ensure retinal image stability (10). VORS requires the subject to suppress their innate VOR while fixating on a target moving with the head (11). These two functions represent the “enhancement” and “suppression” arms of visual-vestibular interaction.

A pioneering study by Arriaga et al. (12) first reported elevated VVOR gain as the most common vestibular test abnormality in VM (71% vs. 5% in controls), proposing it might stem from a “hypersensitive” state of visual-vestibular integration. But, subsequent work found similar elevations in other vestibular disorders (13), questioning its specificity. Conversely, a case report described isolated VORS impairment with VM-like symptoms (14), suggesting that VORS deficits may more directly reflect central dysfunction. However, the prevalence of this finding in the broader VM population remains unverified.

No study has simultaneously characterized both VVOR and VORS in the same VM cohort. Thus, it is unclear whether VM manifests as enhanced integration (elevated VVOR), insufficient suppression (elevated VORS), or both, and which metric better reflects disease-related central vestibular dysfunction. We hypothesized that VM patients would show both elevated VVOR and VORS gains compared to healthy controls, but that VORS impairment—reflecting defective cerebellar inhibitory control—would be more specifically associated with VM risk, whereas elevated VVOR would represent a less specific adaptive response.

To test our hypothesis, this study systematically evaluated VVOR and VORS functions within the same VM cohort. Specifically, we aimed to: (1) characterize alterations in VVOR and VORS in VM patients; (2) explore combinatorial patterns of abnormalities to define potential functional subtypes; and (3) provide evidence for central mechanisms of VM. Methods included video-oculography during 0.5 Hz passive head rotation in 57 VM patients and 30 controls, with group comparisons and dose–response analyses adjusting for confounders.

Previous studies have evaluated vestibulo-ocular reflex function in vestibular migraine using high-frequency head impulse paradigms, including HIMP and SHIMP. These studies have provided important evidence that conventional VOR gain may remain largely preserved in many patients with vestibular migraine, while subtle abnormalities may be reflected by parameters such as anti-compensatory saccades. However, HIMP and SHIMP primarily assess high-acceleration semicircular canal function, during which visual feedback is minimized. In contrast, VVOR and VORS assess gaze stabilization and VOR suppression during low-frequency head motion, a condition in which visual input substantially contributes to vestibular control. Because vestibular migraine is frequently associated with visual motion sensitivity and impaired visual–vestibular integration, low-frequency VVOR and VORS testing may provide complementary information that is not captured by high-frequency VOR gain measurements alone. Therefore, the present study aimed to characterize VVOR and VORS function in patients with vestibular migraine and to explore whether these visually dependent vestibular parameters are associated with VM status.

## Subjects and methods

### Subjects

This retrospective case–control study enrolled patients diagnosed with VM who were hospitalized in the Department of Neurology, Beijing Luhe Hospital, Capital Medical University, between January 2024 and February 2025. The diagnostic criteria adhered to the 2022 consensus document of the Bárány Society and the International Headache Society (15): (1) At least five episodes of moderate or severe vestibular symptoms lasting between 5 min and 72 h; (2) Current or past history of migraine with or without aura according to the International Classification of Headache Disorders (ICHD-3); (3) At least 50% of vestibular episodes accompanied by at least one of the following migrainous features: headache fulfilling at least two criteria (unilateral location, pulsating quality, moderate/severe intensity, aggravation by routine physical activity), photophobia and phonophobia, or visual aura; (4) Not better accounted for by another vestibular or ICHD diagnosis. All patients underwent both VVOR and VORS testing after admission. A total of 57 eligible patients were included, comprising 20 males and 37 females, with an age range of 61.50–79.00 years. Individuals who underwent health check-ups during the same period and completed VVOR and VORS testing served as the control group. The study was approved by the Ethics Committee of Beijing Luhe Hospital, Capital Medical University (Approval No.2026-LHKY-019-01), and all participants provided written informed consent.

### Exclusion of other vestibular and central disorders

Hearing screening, and positional testing, including Dix–Hallpike and roll tests when clinically indicated, were performed to exclude benign paroxysmal positional vertigo, Ménière’s disease, vestibular neuritis, middle ear disease, and other peripheral vestibular disorders. Cranial magnetic resonance imaging was performed to exclude central causes, including cerebellar or brainstem lesions, demyelinating disease, posterior fossa tumors, and other structural abnormalities. Participants with confirmed or suspected alternative vestibular or central diagnoses were excluded from the study. Audiologic evaluation, including pure-tone audiometry and tympanometry, was performed when available to further assess hearing status and exclude significant middle ear or cochlear pathology.

### Clinical data

The following data were extracted from the hospital’s electronic medical record system: (1) Demographic characteristics: sex, age, medical history, family history; (2) Clinical features: disease duration, attack characteristics, accompanying symptoms; (3) Comorbidities: hypertension, diabetes, hyperlipidemia, osteoporosis, migraine, insomnia, etc.; (4) Previous vertigo history: first episode or recurrence.

### Vestibular function tests

The VVOR and VORS testing were completed within 1 week after admission/enrollment. Both tests were performed consecutively during the same testing session on the same day using the same video-oculography system. All VM patients were tested during the interictal period, at least 2–3 days after the most recent vertigo or headache attack. No participant reported active vertigo, headache, nausea, or other residual attack-related symptoms on the day of VVOR and VORS testing. Tests were performed using the VertiGoggles video-oculography system. During testing, participants were seated comfortably, with their eyes approximately 1.2 m from a display screen. Passive horizontal head rotations were applied by the examiner. Eye movements were recorded throughout by the system, and gain values were calculated to assess visual-vestibular integration. The specific test paradigms were as follows:

(1) VVOR test: Participants fixated on a stationary target at the center of the screen in front of them. The examiner passively rotated the participant’s head in the horizontal plane at a frequency of 0.5 Hz and an amplitude of approximately ±15°. This paradigm requires the visual and vestibular systems to cooperate to maintain gaze stability.

(2) VORS test: Participants wore a mask that blocked ambient light. The only visual stimulus was a fixation light attached to the inside of the mask, which moved precisely with the head. The examiner applied passive head rotations with the same frequency and amplitude (0.5 Hz, ±15°). This paradigm requires the brain to suppress the VOR to maintain fixation on a head-fixed target during head motion.

### Statistical analysis

Data were analyzed using SPSS 26.0 and R software (version 4.3.0). A *p*-value < 0.05 was considered statistically significant. Categorical variables were presented as numbers and percentages [*n*(%)]and compared between groups using the chi-square test or Fisher’s exact test. Continuous variables were first tested for normality and homogeneity of variance. Variables with a normal distribution and homogeneous variance were expressed as mean ± standard deviation (^−^*x* ± s) and compared using the independent samples *t*-test. Non-normally distributed variables were expressed as median (interquartile range) [*M* (P25, P75)] and compared using the Mann–Whitney *U* test.

In our study, we applied restricted cubic splines (RCS) to flexibly model the potentially nonlinear relationships between VVOR, VORS, and the risk of vestibular migraine, while simultaneously avoiding the extrapolation biases inherent in purely parametric models. Given that many biomedical relationships are fundamentally nonlinear, forcing them into a linear framework may lead to model misspecification and obscure true exposure–response patterns.

#### Non-linearity and restricted cubic splines (RCS)

To flexibly model potential nonlinear relationships, restricted cubic splines (RCS) with four knots were used, placed at the 5th, 35th, 65th, and 95th percentiles of the predictor distribution. The presence of nonlinearity was formally tested using the likelihood ratio test, comparing the spline model with a linear model; a *p*-value for non-linearity < 0.05 indicated a significant non-linear association.

#### Dichotomization strategy for continuous variables

For each continuous predictor (e.g., VVOR and VORS), we plotted the estimated odds ratios (ORs) with 95% confidence intervals (CIs) against the predictor values to visualize the dose–response curve and identify potential risk thresholds. Based on the RCS results, the following decision rule was applied to dichotomize continuous variables for subsequent binary logistic regression:

(1) If a significant nonlinear relationship was detected and a clear threshold was visually identifiable, the variable was dichotomized at the abscissa of the intersection between the fitted dose–response curve and the null line (OR = 1).

(2) If no significant nonlinearity was found (i.e., *P* for non-linearity ≥ 0.05), the variable was dichotomized using the value corresponding to an OR of 1 as the cut-off point.

The potential risk threshold was identified from the RCS dose–response curve. For an approximately linear association, the value corresponding to the intersection between the fitted curve and the null line (OR = 1) was used as the cut-off point. For a nonlinear association, the visually identifiable threshold or inflection point at which the risk began to increase substantially was selected for dichotomization. Age, mean VVOR gain, and mean VORS gain were included as candidate risk-related variables; mean values were used to avoid collinearity between left- and right-sided measurements.

#### Binary logistic regression

After dichotomization, binary logistic regression models were used to assess the association between each dichotomized variable and vestibular migraine (VM) risk. Odds ratios and 95% confidence intervals were calculated with adjustment for potential confounders, such as age.

Conventional statistical analyses were performed using SPSS. RCS modeling, nonlinearity tests, and graphical visualization were conducted in R using packages such as ‘rms’ and ‘ggplot2’.

## Results

### Representative eye movement recordings

**Figure 1:**
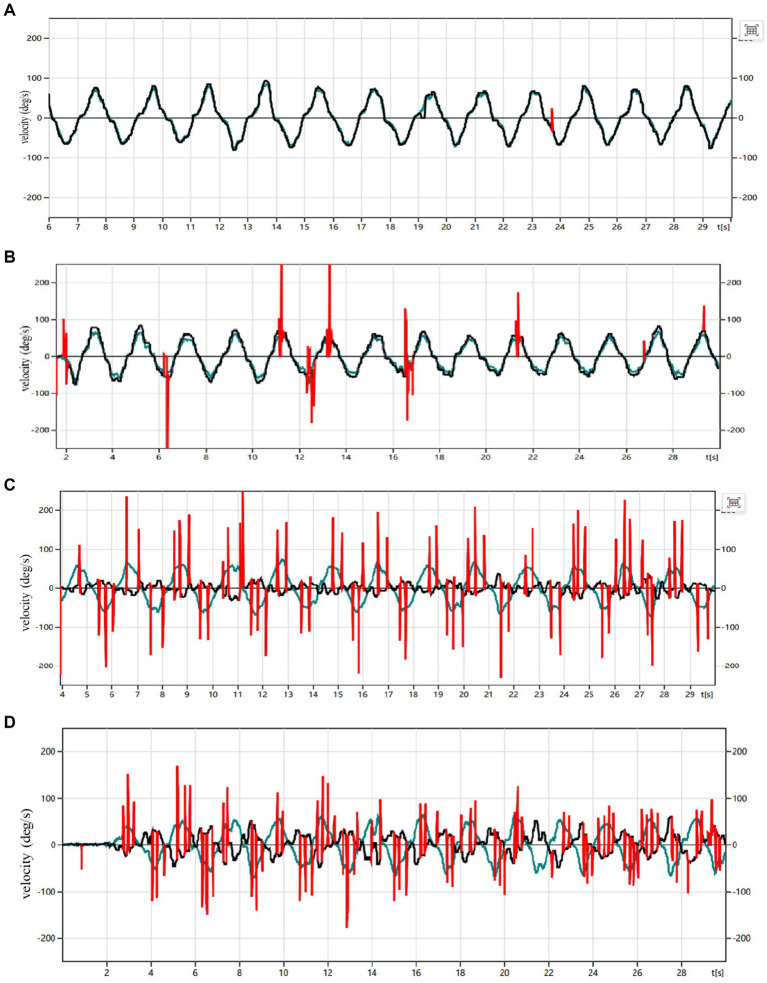
**(A)** Healthy control – VVOR task. **(B)** Patient with VM–VVOR task. **(C)** Healthy control – VORS task. **(D)** Patient with VM– VORS task.

Representative eye movement recordings from a healthy control and a patient with VM during the VVOR and VORS tasks are presented in Figure 1, the green trace represents head velocity over time, and the black trace represents eye velocity over time. During the VVOR task (Figures 1A,B), the healthy control exhibited stable fixation with minimal or no corrective saccades (red traces), reflecting intact visual–vestibular integration. In contrast, the patient with VM frequently showed an increased number of corrective saccades during head movements to either side, indicating impaired central integration of vestibular and visual signals. During the VORS task (Figures 1C,D), the healthy control demonstrated effective VOR suppression, characterized by minimal residual eye movements with only occasional appropriate corrective saccades, whereas the patient with VM showed markedly impaired suppression, with substantially larger residual eye movements and an increased number of corrective saccades.

### Comparison of age, sex, VVOR, and VORS between VM patients and controls

A total of 57 VM patients and 30 healthy controls were included. The VM group comprised 37 females (64.91%), with an age range of 61.50–79.00 years. The control group included 20 females (66.67%), with an age range of 57.75–74.75 years. No statistically significant differences were found between the two groups in age (*Z* = −1.962, *p* = 0.050) or sex distribution (*χ*^2^ = 0.027, *p* = 0.870), indicating good comparability. A comparison of age, sex, VVOR, and VORS between the two groups is presented in Table 1.

**Table 1:** Comparison of age, sex, VVOR, and VORS between VM patients and controls.

Variable	VM group (*n* = 57)	Control group (*n* = 30)	*P*-value
Age (years)	73.00(61.50–79.00)	64.00 (57.75–74.75)	0.050
Sex (female)	37 (64.91%)	20 (66.67%)	0.870
VVOR right	1.37 ± 0.20	1.24 ± 0.20	0.003^*^
VVOR left	1.35 ± 0.19	1.23 ± 0.17	0.006^*^
VVOR (mean)	1.4 ± 0.2	1.2 ± 0.2	0.003^*^
VORS right	0.5 ± 0.2	0.2 ± 0.1	<0.001^*^
VORS left	0.5 ± 0.2	0.3 ± 0.1	<0.001^*^
VORS (mean)	0.5 ± 0.2	0.2 ± 0.1	<0.001^*^

Data are presented as Median (IQR), *n*(%), or mean ± SD. * indicates *p* < 0.05.

### Independent risk factors for vestibular migraine

Age, mean VVOR gain, and mean VORS gain were included as candidate risk-related variables. VM patients showed significantly higher mean VVOR and VORS gains than controls. Representative videonystagmography recordings showed more corrective saccades during VVOR testing and larger residual eye movements with increased corrective saccades during VORS testing in VM patients, supporting impaired visual–vestibular integration and VOR suppression. RCS analysis identified 1.32 as the VVOR cut-off and 0.39 as the VORS threshold for subsequent risk analysis.

**Figure 2:**
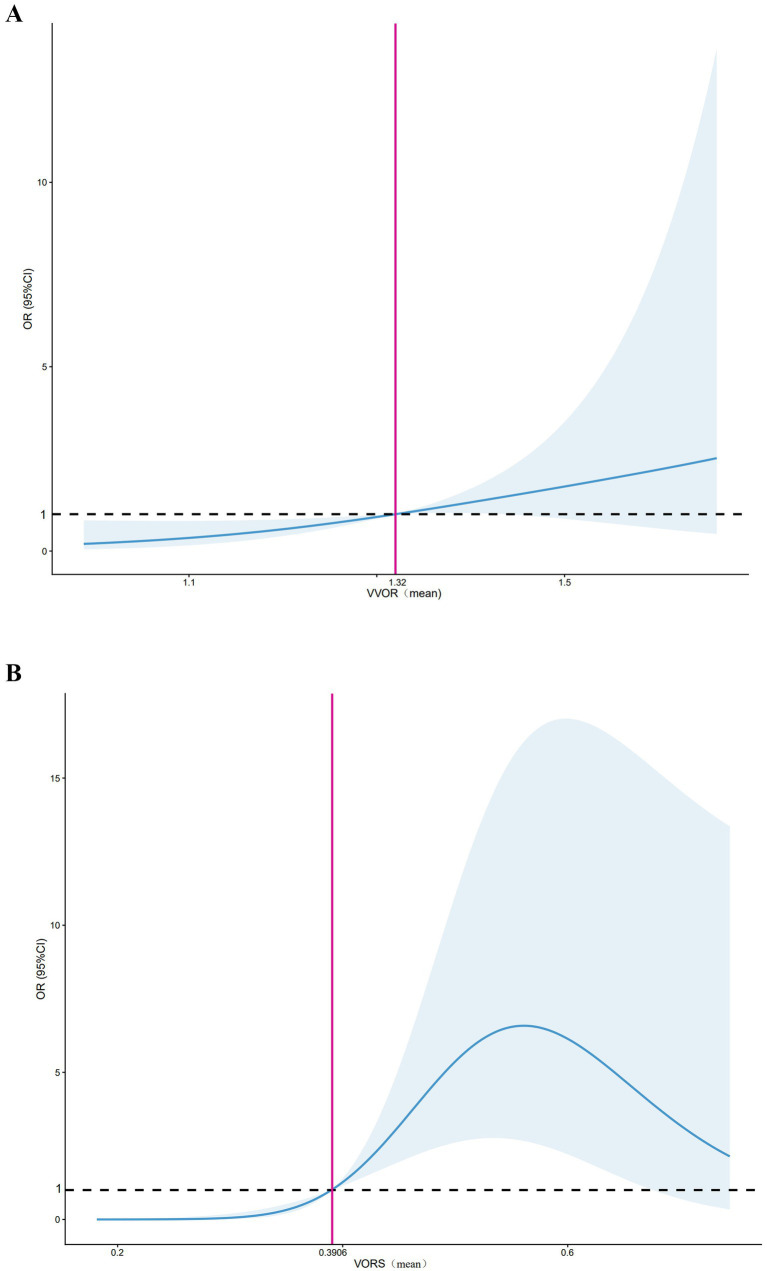
**(A)** RCS curve for mean VVOR *(Image placeholder: RCS curve showing linear association between VVOR and VM risk)*. **(B)** RCS curve for mean VORS *(Image placeholder: RCS curve showing S-shaped nonlinear association between VORS and VM risk)*.

To avoid potential collinearity between left and right sides measurements, the mean values of VVOR and VORS were used as analytical indicators. Restricted cubic spline (RCS) regression was employed to assess the relationship between these vestibular function indices and the risk of VM. RCS analysis revealed a linear association between mean VVOR gain and VM risk (non-linearity *p* = 0.5629). The risk of VM showed a continuous increasing trend with higher VVOR values. Using the point on the curve corresponding to an OR of 1 as a reference, the risk began to increase notably when the mean VVOR gain exceeded 1.32 (see Figure 2A). In contrast, a significant nonlinear association was found between mean VORS gain and VM risk (nonlinearity *p* = 0.0001). The dose–response curve exhibited an S-shape: the risk increased slowly at relatively low VORS levels (approximately <0.39), then accelerated sharply within the range of 0.39 to 0.60, before plateauing again when VORS exceeded 0.60 (see Figure 2B).

### Comparative analysis of vestibular migraine risk

**Figure 3:**
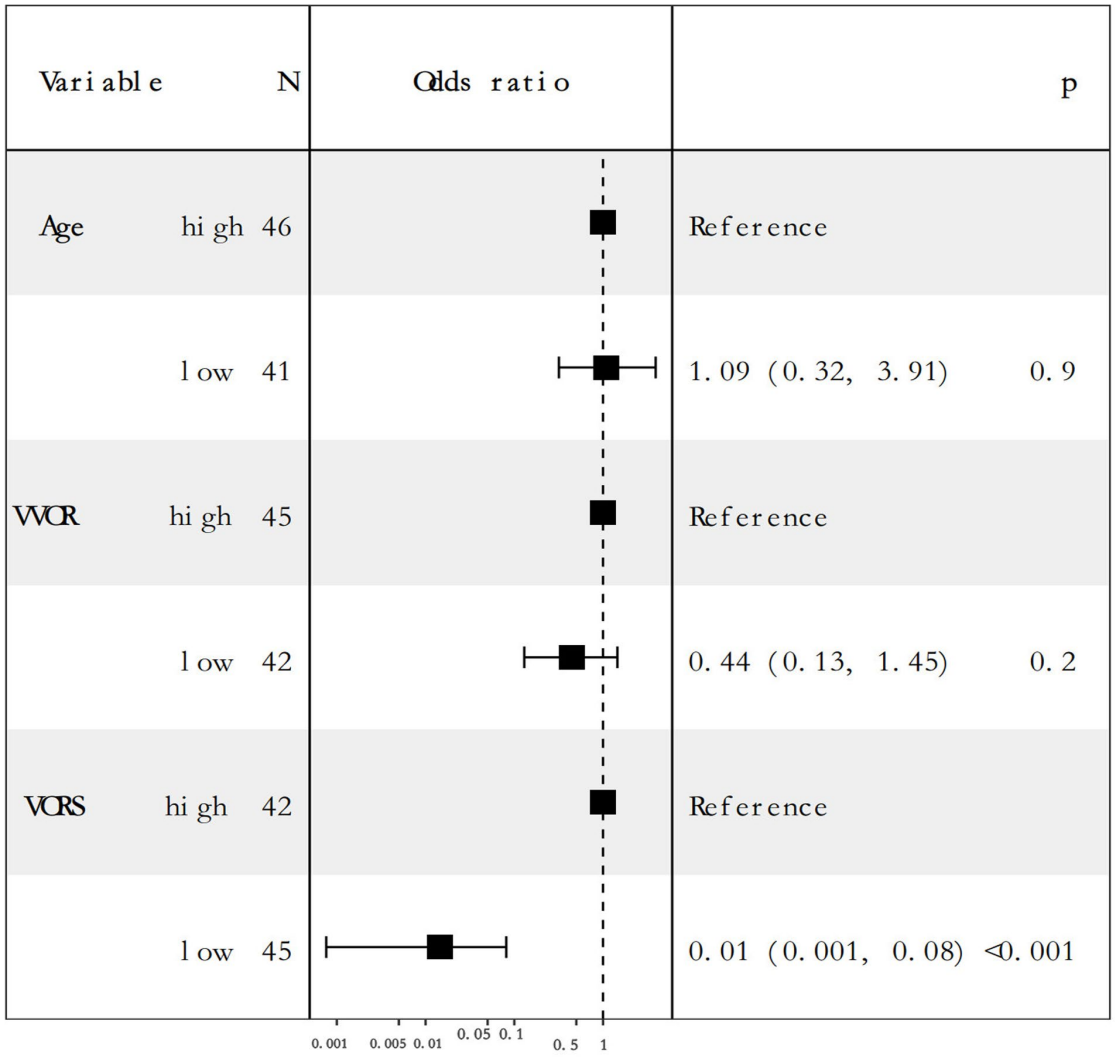
Forest plot showing the association of dichotomized variables with VM risk [*Age (cut-off 68 years), VVOR (cut-off 1.32), and VORS (cut-off 0.39) were analyzed using binary logistic regression. High VORS (≥0.39) was significantly associated with increased VM risk*].

To provide clinically intuitive risk estimates, continuous variables were dichotomized based on the RCS results: for VVOR (linear relationship), the cut-off was the value corresponding to OR = 1 (1.32); for VORS (non-linear relationship with a threshold), the cut-off was the identified inflection point (0.39). Binary logistic regression including age, mean VVOR gain, and mean VORS gain was then performed to identify the factors associated with VM risk. Age was included because of the older age distribution of the cohort, while VVOR and VORS were included as the key vestibular function parameters reflecting visual–vestibular enhancement and VOR suppression, respectively. Older age was not significantly associated with VM risk. High VVOR gain (≥1.32) showed only a non-significant trend after adjustment. In contrast, VORS gain was the strongest risk-related vestibular factor. Compared with the high VORS group (≥0.39), the low VORS group (<0.39) showed a markedly lower likelihood of VM (OR = 0.01, 95% CI: 0.001–0.08, *p* < 0.001), indicating that elevated VORS gain is strongly associated with increased VM risk (see Figure 3).

## Discussion

This study presents the first systematic evaluation of both the “enhancement” (VVOR) and “suppression” (VORS) pathways of visual-vestibular integration within a single cohort of patients with vestibular migraine (VM). Our most striking finding concerns VORS. While the control group exhibited a mean VORS gain of approximately 0.2(SD 0.1), reflecting physiological incomplete suppression, the VM group showed a significantly elevated mean gain of approximately 0.5(SD 0.2, *P* < 0.001). Furthermore, the association between VORS gain and VM risk was distinctly non-linear (*p* = 0.0001), with risk accelerating most rapidly within the 0.39–0.60 range, identifying a critical “risk window” for this parameter. A high VORS gain (≥0.39) was strongly associated with VM (OR = 0.01, 95% CI: 0.001–0.08, *p* < 0.001), establishing VORS impairment as the more clinically relevant abnormality. This phenomenon has rarely been reported in previous VM literature and may point toward a more specific pathophysiological mechanism.

To provide clinically intuitive risk estimates, Binary logistic regression was then used to calculate the association between these dichotomized variables and VM risk, presented as odds ratios with 95% confidence intervals (see Figure 3). Compared to the younger age group (dichotomized at 68 years), the older group did not show a significantly increased risk (OR = 1.09, 95% CI = 0.32–3.91, *p* = 0.9). Compared to the low VVOR group (<1.32), the high VVOR group (≥1.32) showed a trend toward increased risk that was not statistically significant (OR = 0.44, 95% CI = 0.13–1.45, *p* = 0.2). Importantly, compared to the low VORS group (<0.39), the high VORS group (≥0.39) demonstrated a significantly and substantially increased risk of VM (OR = 0.01, 95% CI = 0.001–0.08, *p* < 0.001). Note: the RCS curve clearly shows increasing risk with higher VORS. The key finding is the statistically significant association between high VORS (≥0.39) and VM.

Research by Ramos et al. (16) in patients with vestibular hypofunction, using the same frequency (0.5 Hz), provides crucial context for interpreting VORS. They elucidated that the VORS test requires active suppression of the inherent VOR to fixate on a head-fixed target during head motion. The markedly elevated gain (~0.5) in our VM patients indicates a pathologically enhanced inability to suppress the VOR, that is, during head movement, the cerebellum struggles to attenuate vestibular-driven eye movements, allowing a larger component of the VOR to “break through.” This deficit carries strong pathophysiological significance (17). Normal VORS execution critically depends on precise inhibitory control by the cerebellum over the vestibular nuclei. A case report by Migliaccio and Watson (14) already linked isolated VORS impairment, VM-like symptoms, and lesions in the dorsal cerebellar vermis. Thus, our finding of VORS suppression deficits in VM patients may directly reflect dysfunction within cerebello-vestibular inhibitory circuits, specifically an impairment in the cerebellum’s ability to exert gain control and selectively inhibit sensory input. This aligns with current theories positioning VM as a disorder involving central sensitization, abnormal multisensory integration, and cerebellar modulation dysfunction (7, 18, 19). The observed plateau in risk acceleration beyond a VORS gain of 0.60 could reflect that at such extremely high levels, patients may be at increased risk for other vestibular conditions (20), thereby appearing as a risk plateau within the current diagnostic framework for VM. Conversely, the VORS gain threshold of 0.39—which yielded an odds ratio of 0.01, indicating that a gain below this level is highly protective—may signify a critical point where cerebellar compensatory mechanisms begin to fail or pathological changes reach a clinically significant level. Studies in ischemic stroke have identified futile reperfusion and reperfusion injury as major determinants of poor outcome, mediated by microvascular thrombo-inflammation (21–25). These findings raise the possibility that similar mechanisms within cerebellar-vestibular circuits may underlie VORS suppression deficits in vestibular migraine.

Regarding VVOR, the control group exhibited a mean gain of approximately 1.2 (SD 0.2), exceeding the conventional normal upper limit of 1.0 reported in some earlier studies [e.g., Arriaga et al. (12)]. The VM group showed a significantly higher gain (1.4 ± 0.2 vs. 1.2 ± 0.2, *p* = 0.003). This discrepancy in absolute values is likely attributable to two key factors. First, test frequency is a fundamental determinant. Many previous studies (e.g., Arriaga and Kim) employed low-frequency rotations (0.04–0.08 Hz), designed to assess visual compensation for slow head movements. In contrast, consistent with Ramos et al. (16), our study utilized a frequency of 0.5 Hz. As Rey-Martinez et al. (26) demonstrated, VVOR gain is frequency-dependent. The higher baseline gains observed in our study may partially reflect the greater contribution of smooth pursuit and optokinetic reflexes to ocular stability at this relatively low-to-mid frequency (0.5 Hz), resulting in a systematically higher calculated overall gain. Secondly, the distinctively older age profile of our cohort is a crucial explanatory factor. The median age of our VM group (73 years) was substantially higher than that in previous studies (e.g., Arriaga/Kim cohorts ~43–51 years). Although Rey-Martinez et al. (26) found no significant association between age and VVOR gain at their specific tested frequency, advanced age is generally accompanied by physiological declines in vestibular function. This may trigger compensatory CNS adaptations, potentially upweighting the reliance on visual cues to maintain stability. The potential influence of age-related vestibular decline should also be considered. Presbystasis is commonly associated with reduced vestibular function and impaired gaze stabilization in older adults. In the present study, however, VM patients showed increased VVOR gain compared with controls, which is opposite to the expected direction of age-related vestibular decline. In addition, the VM and control groups were age-matched, with no statistically significant difference in age, and age was adjusted for in the multivariate analyses. Therefore, although age-related vestibular changes cannot be completely excluded, presbystasis alone is unlikely to explain the elevated VVOR gain observed in VM patients. Critically, despite being elevated in VM patients, VVOR gain showed a linear relationship with disease risk (nonlinearity *p* = 0.5629), and a high gain (≥1.32) was not independently associated with VM after confounder adjustment (OR = 0.44, 95% CI: 0.13–1.45, *p* = 0.2). Together with Kim’s findings (13), that elevated VVOR gain occurs across various vestibular disorders (VM, Meniere’s, BPPV), our results reinforce the view that elevated VVOR gain is a non-specific, cross-disease adaptive response to vestibular dysfunction or unreliable sensory input. Its value as a specific diagnostic marker for VM is limited, and clinical interpretation must rigorously consider the testing frequency and the patient’s age. Despite its limited specificity, elevated VVOR gain in clinical practice, especially in patients with atypical histories or unremarkable routine tests, can still serve as a clue prompting clinicians to consider underlying VM or a state of general vestibular system dysfunction.

Our study demonstrates the coexistence of elevated VVOR gain and VORS suppression deficits in VM patients, yet their association patterns with the disease differ fundamentally, suggesting that the “enhancement” and “suppression” pathways of visual-vestibular integration may be differentially affected. We propose the following explanatory framework: Elevated VVOR gain likely represents a non-specific “state” change, reflecting CNS upweighting of visual cues to cope with sensory conflict or uncertainty (e.g., from trigeminovascular activation, cortical spreading depression, or sensory hypersensitivity) (27–29), ultimately aiming to minimize retinal slip. In contrast, the VORS suppression deficit—characterized by a threshold-dependent risk profile and strong independent association with VM—may signify a more “trait-like” dysfunction of a specific neural circuit: impaired cerebellar inhibitory control over the vestibular reflex. This could represent a core pathophysiological mechanism, at least in a subgroup of VM patients.

Based on combinatorial patterns of VVOR and VORS abnormalities (e.g., isolated VORS deficit, predominant VVOR enhancement, mixed dysfunction), future research could establish more physiologically grounded endophenotypes of VM. Such subtyping may help explain the clinical heterogeneity of VM (e.g., visual dependence vs. self-motion sensitivity) and enable individualized treatment strategies, including tailored vestibular rehabilitation (30) or pharmacotherapies targeting specific cerebellar functions.

In conclusion, this study provides the first simultaneous evaluation of the enhancement (VVOR) and suppression (VORS) pathways of visual-vestibular integration in a single VM cohort, revealing that while VM patients exhibit both elevated VVOR gain and impaired VORS suppression, their disease associations differ fundamentally. VORS suppression deficit demonstrates a threshold-dependent, nonlinear relationship with VM risk (nonlinearity *p* = 0.0001), with a gain ≥0.39 strongly and independently associated with VM (OR = 0.01, *p* < 0.001), whereas elevated VVOR gain shows a linear relationship (*p* = 0.5629) and loses independent association after confounder adjustment (OR = 0.44, *p* = 0.2). These distinct patterns suggest divergent mechanisms: elevated VVOR gain likely represents a non-specific “state” change reflecting CNS upweighting of visual cues, while VORS suppression deficit may signify a more “trait-like” dysfunction of impaired cerebellar inhibitory control over the vestibular reflex—potentially a core pathophysiological mechanism in at least a VM subgroup. Clinically, VORS gain ≥0.39 emerges as a more specific indicator of VM-related central dysfunction than VVOR, supporting its potential role in mechanistic subtyping and targeted vestibular rehabilitation.

### Limitations and future directions

Several limitations of this study should be acknowledged. The retrospective, single-center design with a relatively limited sample size and the absence of a disease control group (e.g., Meniere’s disease, BPPV) preclude direct assessment of the diagnostic specificity of VVOR and VORS abnormalities for VM versus other vestibular disorders. Additionally, the inclusion of only hospitalized patients may introduce selection bias toward more severe cases. Future multicenter, prospective studies with larger, more representative samples and appropriate control groups are needed to validate the VORS threshold effect and its discriminative power. Additionally, Studies incorporating longer standardized symptom-free intervals may further validate these findings. Integrating functional neuroimaging (e.g., fMRI, DTI) could further elucidate the neural circuitry underlying these findings and enable longitudinal observation of their relationship with clinical evolution and treatment response.

## Data Availability

The raw data supporting the conclusions of this article will be made available by the authors, without undue reservation.
